# Supplementary material for: Electronic Nose Sensor Drift Affects Diagnostic Reliability and Accuracy of Disease-Specific Algorithms
Source: Sensors (Basel). 2022 Nov 28;22(23):9246. doi: 10.3390/s22239246 (PMC9740993; doi:10.3390/s22239246)
Supplement: Supplementary file 1 [file sensors-22-09246-s001.zip › sensors-1903625-supplementary.pdf]

Supplementary Figure S1. A schematic image of the eletronic nose set-up.

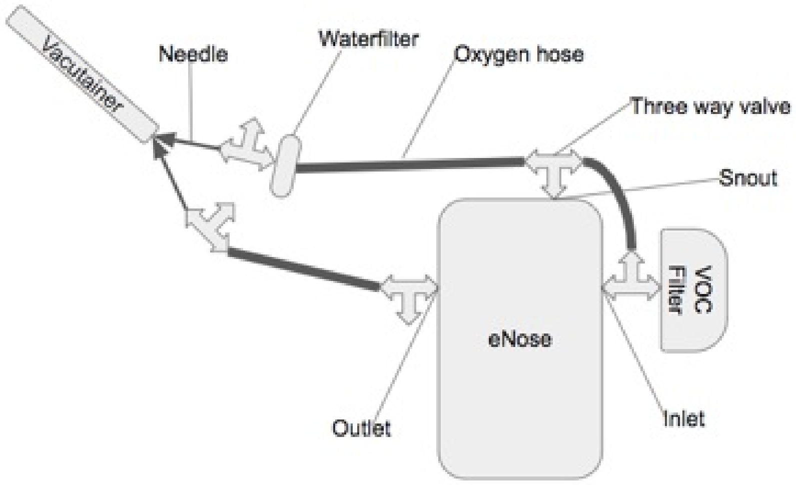

Supplementary Figure S1. Samples were measured using an eNose (Cyranose 320®). The vacutainer is connected to the air-tight loop system of the Cyranose 320® by piercing two needles into the cap of the vacutainer. These needles are attached to two oxygen hoses which are connected to the snout and outlet of the Cyranose 320® by three-way valves. Before the actual VOC analysis is performed, three-way valves are used to create a closed loop between the VOC filter, eNose snout and outlet. Sensors are purged for 90 seconds with filtered ambient air passing the VOC filter to detach the residual VOCs from the sensors. Then, a baseline reference signal is obtained using the filtered air. Subsequently, three-way valves are rotated, creating a closed loop with the vacutainer, eNose snout and outlet to perform the actual measurement for 60 seconds. This loop ensures that ambient air does not dilute VOCs passing the eNose sensors. Then, valves are switched to starting position to allow for another purging of the inlet for 15 seconds and purging of the sensors for 90 seconds, to remove the VOCs from the sensors for the next analysis. After each sample analysis the needles, tubes, connectors and three-way valves are replaced to prevent contamination.

Supplementary Figure S2. Box plots of prediction errors.

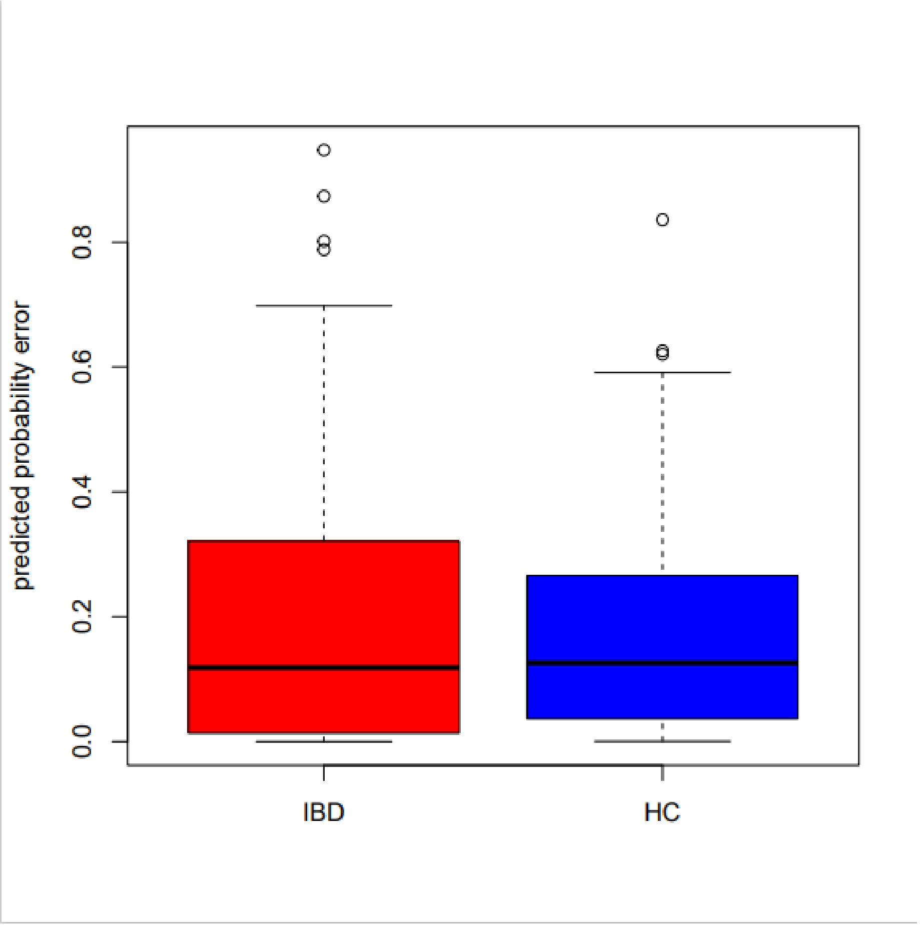

Supplementary Table S1. Disease behaviour and localisation of patients with inflammatory bowel disease.

	Inflammatory bowel disease	
	Crohn's disease (n=24)	Ulcerative colitis (n=39)
Age at diagnosis %, (CD + UC)		
A1 <16	6 (25.0%)	1 (2.6%)
A2 = 17-40	7 (29.2%)	20 (51.3%)
A3 >40	11 (45.8%)	18 (46.2%)
Disease location (CD)		
L1 ileal	8 (33.3%)	N.A.
L2 colonic	6 (25.0%)	N.A.
L3 ileocolonic	9 (37.5%)	N.A.
L4 proximal	-	N.A.
Disease phenotype (CD)		
B1 non-stricturing/non-penetrating	16 (66.7%)	N.A.
B2 stricturing	5 (20.8%)	N.A.
B3 penetrating	2 (8.3%)	N.A.
Perianal abnormalities (CD)		
P1 no	18 (75.0%)	N.A.
P2 yes	5 (20.8%)	N.A.
Upper GI involvement (CD)		
U1 no	23 (95.8%)	N.A.
U2 yes	1 (4.2%)	N.A.
Extent (UC)		
E1 rectum	N.A.	5 (12.8%)
E2 left sided colitis	N.A.	16 (41.0%)
E3 pancolitis	N.A.	16 (41.0%)
Type of endoscopy		
Colonoscopy	23 (95.8%)	36 (92.3%)

Sigmoidoscopy	1 (4.2%)	3 (7.7%)
Surgery		
Ileocaecal resection	6 (25.0%)	-
(partial) colectomy	-	1 (2.6%)
Pouch	1 (4.2%)	1 (2.6%)

Based on the Montreal score at inclusion. Abbreviations: n, number; CD, Crohn's Disease; UC, Ulcerative Colitis; NA, not applicable; GI, gastrointestinal.

Supplementary Table S2. Endoscopic assessment of patients with inflammatory bowel disease.

	Inflammatory bowel disease	
	Crohn's disease (n=24)	ulcerative colitis (n=39)
Van der Heide score		
Mucosal colour		
Normal	13 (54.2%)	15 (38.5%)
Red	10 (41.7%)	23 (59.0%)
Deeply red	-	-
Vascular pattern		
Normal	15 (62.5%)	14 (35.9%)
Partially absent	6 (25.0%)	10 (25.6%)
Absent	2 (8.3%)	14 (35.9%)
Friability		
Normal	17 (70.8%)	29 (74.4%)
Slight	5 (20.8%)	8 (20.5%)
Severe	1 (4.0%)	1 (2.6%)
Granularity		
Absent	19 (79.2%)	17 (43.6%)
Fine	2 (8.3%)	18 (46.2%)
Coarse	2 (8.3%)	3 (7.7%)

Rectal valves		
Sharp	21 (87.5%)	31 (79.5%)
Swollen	1 (4.2%)	6 (15.4%)
Absent	-	-
Extensibility		
Normal	16 (66.7%)	34 (87.5%)
Contracted	4 (16.7%)	4 (10.3%)
Rigid	3 (12.5%)	-
Lymphoid nodularity		
Absent	24 (100%)	39 (100%)
Some	-	-
Multiple	-	-
Ulcers		
Absent	8 (33.3%)	35 (89.7%)
Few	11 (45.8%)	2 (5.1%)
Multiple	4 (16.7%)	1 (2.6%)
Spontaneous bleeding		
Absent	21 (87.5%)	33 (84.6%)
Minimal	2 (8.3%)	5 (12.8%)
Severe	-	-
Mucopurulent exudate		
Absent	15 (62.5%)	27 (69.2%)
Some	8 (33.3%)	5 (12.8%)
Abundant	-	6 (15.4%)
Mayo endoscopic score		
Mayo 0 (normal/inactive)	N.A.	16 (41.0%)

Mayo 1 (mild disease)	N.A.	9 (23.1%)
Mayo 2 (moderate disease)	N.A.	8 (20.5%)
Mayo 3 (severe disease)	N.A.	3 (7.7%)
Rutgeerts' postoperative score (n=6)		
I0 no lesions	2	N.A.
I1 <5 aftous ulcers	-	N.A.
I2 >5 aftous ulcers	3	N.A.
I3 diffuse aftous ileitis	1	N.A.
I4 diffuse inflammation	-	N.A.
Disease activity		
Quiescent	5 (20.8)	17 (43.6%)
Active	19 (79.2)	22 (56.4%)

Abbreviations: n, number; CD, Crohn's Disease; UC, Ulcerative Colitis; NA, not applicable.

Supplementary Table S3. Medication use at time of sample collection per disease activity subgroup.

	CDa (n=19)	CDr (n=5)	UCa (n=22)	UCr (n=17)
Aminosalicylates (n, [%])	0 [0.0]	1 [20.0]	14 [63.6]	12 [70.6]
Corticosteroids (n, [%])	2 [10.5]	1 [20.0]	5 [22.7]	1 [5.9]
Thiopurines (n, [%])	6 [31.6]	2 [40.0]	4 [18.2]	6 [35.3]
Methotrexate (n, [%])	3 [15.8]	0 [0.0]	0 [0.0]	0 [0.0]
Anti-TNF (n, [%])	6 [31.6]	1 [20.0]	2 [9.1]	2 [11.8]
Antibiotics (n, [%])	0 [0.0]	0 [0.0]	1 [4.5]	0 [0.0]

Abbreviations: CD, Crohn's disease; UC, ulcerative colitis; a, active disease; r, remission.
